# Multiplex Cytokine Concentration Measurement: How Much Do the Medium and Handling Matter?

**DOI:** 10.1155/2013/890706

**Published:** 2013-09-28

**Authors:** Luke Parkitny, James H. McAuley, Patrick J. Kelly, Flavia Di Pietro, Barbara Cameron, G. Lorimer Moseley

**Affiliations:** ^1^Neuroscience Research Australia, Barker Street, Randwick, NSW 2031, Australia; ^2^Prince of Wales Clinical School, The University of New South Wales, Randwick, NSW 2031, Australia; ^3^Sydney School of Public Health, The University of Sydney, Edward Ford Building A27, Sydney, NSW 2006, Australia; ^4^Centre for Infection and Inflammation Research, School of Medical Sciences, University of New South Wales, Sydney, NSW 2052, Australia; ^5^The Sansom Institute for Health Research, University of South Australia, North Terrace, SA 5000, Australia

## Abstract

Cytokine concentrations are thought to be affected by methods of sampling and processing and by storage conditions. In this study we compared 17 cytokine concentrations obtained from plasma and serum at baseline and after a controlled thaw condition. We found that absolute agreement was poor between concentrations of cytokines in plasma and serum, except for MIP1**β**. A thaw condition significantly changed the concentrations of most cytokines, but serum appeared less affected by this than plasma was. Closer examination using Bland-Altman analyses revealed that for each comparison, agreement was moderately good for many cytokine concentrations. This is important because measures of agreement must be interpreted based on the required precision, which may differ between clinical and research demands. We also identified that for some cytokines, the relationship between serum and plasma is affected by concentration, thus advocating for the use of appropriate methods when performing such comparisons in studies such as systematic reviews and meta-analyses.

## 1. Introduction

Cytokines are regulatory proteins involved in the control of cell behaviour [1]. Changes in cytokine concentrations have been associated with normal and pathological states of immune activation. Thus, cytokine levels are frequently measured for diagnostic and research purposes, including in the study of ageing [2], response to exercise [3], psychiatric disorders [4], cancer [5], obesity [6], and pain [7].

Cytokine concentrations are thought to be affected by methods of sampling and processing and by storage conditions. How much these factors influence cytokines helps guide research protocols. Serial sampling from large frozen samples would be precluded by large thaw effects on cytokine concentrations. The pooling of cytokine concentrations during the conduct of a systematic review and meta-analysis [8], would be complicated by significant and nonsystematic differences between cytokine concentrations measured in serum versus plasma analytes. It has been suggested that concentrations of cytokines are different in serum and plasma due to the different biological properties of these media [9, 10]. It has also been suggested that multiplex arrays may be more susceptible to obtaining different cytokine level measurements in serum as compared to plasma than individual ELISA analyses [2]. The impact of processing and storage, particularly the effect of temperature, has been examined in human serum for cytokines including IL-6, sIL-6R, CC-16, and IL-10 [11] and in plasma and serum for cytokines including IL-6 and TNF-*α* [12]. However, most of this work (1) artificially manipulated samples which may not accurately reflect the behaviour of cytokines as found in the blood of clinical samples and (2) did not measure cytokine levels using a large multiplex cytokine panel. Furthermore, to date, research that has attempted to measure agreement between samples has used only a single statistical method, typically a correlation coefficient. However, it has been recommended that multiple approaches should be used to assess agreement because each method has limitations that are addressed by other methods [13].

In the present study, we comprehensively tested the agreement between the measured levels of 17 cytokines in serum and plasma pairs from clinical samples and the agreement between the measured levels of these 17 cytokines before and after controlled freeze-thaw cycles in serum and plasma. We used multiplex assay technology as this method provides a more comprehensive profile of inflammation by simultaneously measuring several cytokines.

## 2. Materials and Methods

### 2.1. Subjects

The subjects were obtained from a study investigating recovery following wrist and hand fractures. Inclusion criteria for that study were unilateral wrist or hand fracture sustained in the last 7–14 days, aged 18–75, and a sufficient understanding of written and verbal English language. Exclusion criteria were any other area of significant injury or pain, inability to participate due to significant psychiatric illness, existing neurological illness, any condition that prevented normal management of the wrist fracture, and pregnancy. All subjects provided written consent prior to participation. The study was approved by the Human Research Ethics Committee of the Northern Hospital Network, New South Wales, Australia, and the Human Research Ethics Committee of The University of New South Wales, Australia.

### 2.2. Sample Collection and Storage

One 8 mL serum separator tube (SST; Vacuette) and one 9 mL tripotassium salt of ethylenediaminetetraacetic acid plasma tube (K3-EDTA; Vacuette) were collected from a vein in the cubital fossa of the uninjured arm of each subject. SST samples were allowed to coagulate for 30 minutes at room temperature prior to centrifugation. EDTA samples were centrifuged within 15 minutes of collection. Centrifugation was performed at 4°C for 15 minutes and the supernatant was aspirated for immediate storage in 200 *μ*L aliquots at −80°C.

### 2.3. Freeze-Thaw Cycles

Samples from 19 consecutive subjects were selected for this study, provided that sufficient aliquots were obtained from the participant for the recovery study. Testing was performed on 38 aliquots (19 serum and 19 plasma). The two experimental conditions included (1) a baseline assay from a sample thawed at room temperature and (2) a single or double additional thaw-freeze cycle prior to the assay. To complete the thaw-freeze cycle, frozen samples were left undisturbed at room temperature until they were visibly thawed plus for an additional 60 minutes. Samples undergoing two thaw-freeze cycles were refrozen at −80°C for 24 hours prior to the second thaw. All thaw-freeze samples were then returned to storage at −80°C until assay. Each participant's sample was subjected to condition 1; 12 participants' samples were also subjected to condition 2.

### 2.4. Assay Procedures

Cytokine quantification was performed in duplicate. Serum and plasma samples were thawed on ice and concentrations of the cytokines IL-1*β*, IL-2, IL-4, IL-5, IL-6, IL-7, IL-8, IL-10, IL-12p70, IL-13, IL-17, G-CSF, GM-CSF, TNF-*α*, and IFN-*γ* and the chemokines MCP-1 and MIP-1*β* were analysed according to the manufacturer's instructions for the bead-based multiplex immunoassay system (BioPlex, BioRad, Hercules, CA). Test samples were incubated with bead mix at room temperature, and then washed three times while retaining the beads. The beads were then incubated with the biotinylated detection antibody mix, washed three times, streptavidin-PE was added, washed again, and finally resuspended in an assay buffer for reading on the BioPlex instrument (Bio-Rad). All assay procedures were performed by investigator B. Cameron who was blinded to the samples and the aims of the study. The final concentration of analytes was calculated using the Bio-Plex Manager v5.0 software package (Bio-Rad). For all statistical analyses, values below the detection threshold of the assay were replaced with the minimal detectable value for the analyte. To assist the interpretation of results, two coefficients of variation (CV%) were calculated for each cytokine and are presented in Table 2. To test the overall precision of the plate, an intra-assay CV% was calculated as the mean of the individual CV% values of all duplicates. We also calculated a low-concentration CV% to reflect the precision of each test at the concentrations most relevant to our samples. This was calculated from the mean of the individual CV% values of all duplicates ranging from the most dilute standard to the standard equivalent to the median of the measured concentrations. As a general rule, CV% values less than 10% were deemed to be acceptable.

### 2.5. Data Analysis and Statistics

Agreement was assessed for assay results between baseline serum and baseline plasma samples, between the baseline and postthaw-freeze samples for serum, and between the baseline and postthaw-freeze samples for plasma. The latter two comparisons examined the stability of cytokine levels following the thaw-freeze condition. Agreement was measured using intraclass correlation coefficients (ICCs) and their 95% confidence intervals (95% CI) based on a two-way mixed-effects model (ICC 3,1) [14], and Bland-Altman analysis [15]. 

The ICC estimates agreement or reliability. In this study, the ICC indicates the degree of consensus between the two cytokine concentrations (e.g., plasma versus serum). The ICC can take a value from 0.00, suggesting no agreement, to a value of 1.00, indicating perfect agreement. The closer the ICC value is to 1.00, the better the agreement, an ICC value over 0.50 suggests reasonable agreement; 0.61–0.75 indicates good agreement, and a value over 0.75 indicates excellent or near perfect agreement [16, 17]. While the ICC provides an easily interpretable statistic, it does not assess systematic bias. Bias, however, can be assessed using a Bland-Altman analysis. 

Bland-Altman analysis involves plotting the difference between the two cytokine concentrations (e.g., plasma minus serum) against the average of the two concentrations; the average is assumed to most closely represent the true value of the concentration. The Bland-Altman plots also show horizontal lines indicating ninety-five percent (95%) limits of agreement which are ±1.96 standard deviations (SD) of the differences between the two concentrations [15, 18]. The two concentrations are considered to be very similar, or in good agreement, if the points and limits of agreement are close to the horizontal line *y* = 0. If the two concentrations are not similar, the points and the limits of agreement will be further away from *y* = 0. Interpreting the limits of agreement is subjective and must be examined in the context of what is considered to be a biologically significant difference. In this paper, we classify agreement as “moderately good” when the limits of agreement are less than the mean concentration of the tested cytokine.

To test for bias, we also calculated the mean of the differences and its 95% CI. If there is no systematic difference between the two concentrations, then the mean of the differences should be close to zero; a value of zero indicates no bias. If the 95% CI for the mean of the differences does not include zero, it suggests a systematic difference (or bias) between the two concentrations.

All analyses were performed using SPSS (v20), with Bland-Altman analyses conducted using a published syntax [19]. A 5% significance level was used.

## 3. Results

### 3.1. Study Subjects

Twenty-six subjects (11 females and 14 males; age range 18–73 years) were enrolled between October 2010 and December 2010 from the Prince of Wales Hospital in Sydney, Australia. Assay testing was performed on January, 25, 2011; samples were stored for a maximum of three months.

### 3.2. Sample Cytokine Agreement

**Figure 1:**
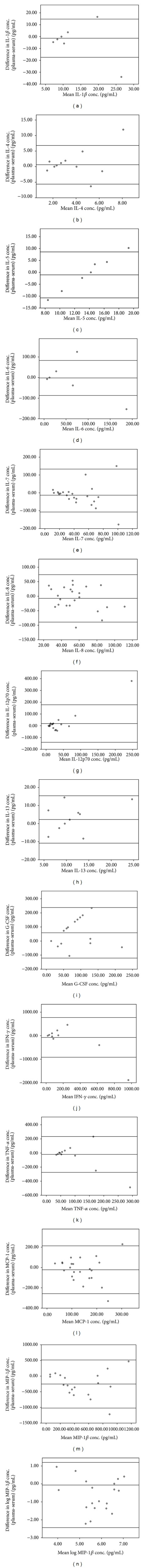


**Figure 2:**
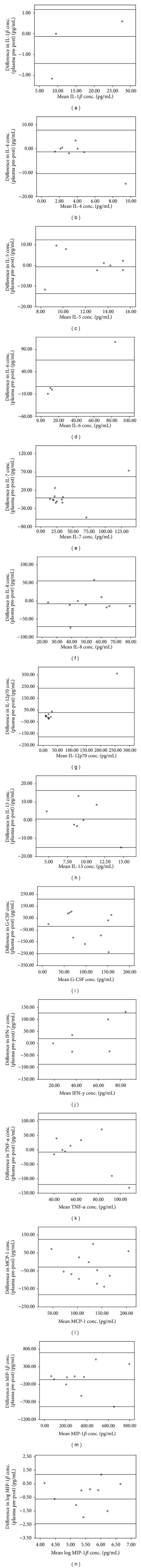


**Figure 3:**
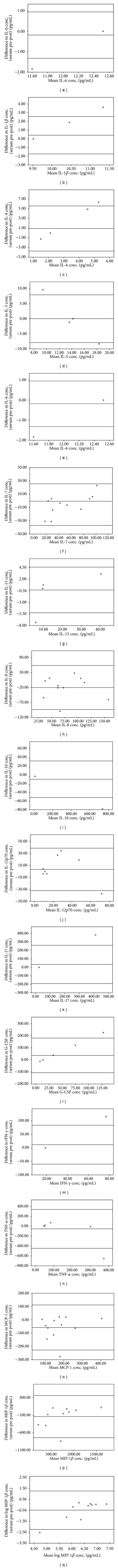


The percentages of serum and plasma samples that were measured as out-of-range (below the assay detection limit) for each factor are presented in Table 1. Due to the substantial proportions of out-of-range values, we did not test for differences between agreements in the single and double thaw-freeze cycle conditions. The results of agreement tests are presented in Table 2 and in Figures 1(a)–3(q).

#### 3.2.1. Agreement between Plasma and Serum at Baseline

Using ICCs, poor agreement was found between plasma and serum in IL-1*β*, IL-4, IL-5, IL-6, IL-7, IL-8, TNF-*α*, IFN-*γ*, IL-12p70, IL-13, IL-17, G-CSF, MCP-1, and MIP-1*β*. IL-2, IL-10, and GM-CSF were not detectable in any plasma samples although they were detected in some serum samples. The concentration of one IL-6 serum sample was found to be very high (5886.12 pg/mL), but its removal did not significantly change the ICC value for this analyte.

Bland-Altman analyses revealed that agreement was moderate for IL-1*β*, IL-5, IL-6, IL-8, and MIP-1*β* and low for IL-4, IL-7, IL-12p70, IL-13, G-CSF, IFN-*γ*, TNF-*α*, and MCP-1. Agreement was improved at low concentrations of: IL-1*β* (<15 pg/mL), IL-4 (<4 pg/mL), IL-6 (<50 pg/mL), IL-7 (<50 pg/mL), IL-12p70 (<100 pg/mL), IFN-*γ* (<200 pg/mL), TNF-*α* (<100 pg/mL), and MCP-1 (<140 pg/mL). A small degree of systematic bias was present for all cytokines and was substantial for G-CSF and MIP-1*β*. 

#### 3.2.2. Agreement between Baseline and Thaw Conditions

In plasma, ICCs indicated poor agreement between the baseline and after a thaw-freeze cycle for IL-4, IL-5, IL-6, IL-8, TNF-*α*, IFN-*γ*, IL-12p70, IL-13, G-CSF, MCP-1, and MIP-1*β*. Only IL-1*β* showed excellent agreement. Bland-Altman analyses demonstrated that agreement was moderate for IL-1*β*, IL-8, IL-13, and MCP-1 but low for IL-4, IL-5, IL-6, IL-7, IL-12p70, IL-13, G-CSF, IFN-*γ*, TNF-*α*, and MIP-1*β*. Agreement was improved at low concentrations of: IL-4 (<4 pg/mL), IL-6 (<20 pg/mL), IL-7 (<30 pg/mL), IL-12p70 (<50 pg/mL), IFN-*γ* (<40 pg/mL), TNF-*α* (<70 pg/mL) and MIP-1*β* (<300 pg/mL). Agreement was high for IL-8 except for 2 outliers, and improved at higher concentrations for IL-5 (>12 pg/mL). A small degree of systematic bias was present for all cytokines. There was insufficient data to produce a Bland-Altman plot for IL-17.

In serum, ICCs suggested poor agreements between the baseline and after a thaw-freeze cycle for IL-1*β*, IL-4, IL-5, IL-6, IL-8, TNF-*α*, IFN-*γ*, IL-12p70, IL-13, G-CSF, MCP-1, and substantial agreements for IL-17 and MIP-1*β*. Excellent agreements were found for samples of IL-7, IL-10, and IL-13. Bland-Altman analyses demonstrated that agreement was moderate for IL-6, IL-7, IL-8, IL-10, IL-13, IFN-*γ*, TNF-*α*, MCP-1, MIP-1*β* and low for IL-1*β*, IL-4, IL-5, IL-12p70, IL-17, and G-CSF. A small degree of systematic bias was present for all cytokines and was substantial for IL-4, IL-8, G-CSF, MCP-1, and MIP-1*β* IL-12p70. Agreement decreased with increasing concentration for IL-1*β*, IL-6, IL-7, G-CSF, TNF-*α*, MCP-1, and MIP-1*β*. 

The Bland-Altman plots showed that the size of the difference of IL-1*β*, IL-6, IL-7, G-CSF, TNF-*α*, MCP-1, and MIP-1*β* increased as the mean concentration increased, suggesting that the concentrations should be analysed after a log transformation. An examination of ICCs and Bland-Altman plots of the log-transformed cytokine concentrations did not change the agreement values except for MIP-1*β*. Following transformation, the ICCs for logged MIP-1*β* were on average much higher: plasma versus serum 0.72 (0.29, 0.89); plasma baseline versus thaw 0.59 (−0.66, 0.90); and serum baseline versus thaw 0.63 (−0.49, 0.91). The Bland-Altman plot for logged MIP-1*β* is also presented in Figure 2(n) showing improved agreement.

## 4. Discussion

We found that the absolute agreements between cytokine concentrations measured using a multiplex assay in serum and plasma were generally poor across all comparisons. A more detailed inspection using Bland-Altman analyses confirmed this, but also revealed that: very good agreement was present for some cytokines only at lower or higher concentrations; some agreements changed with increasing cytokine concentration; and that most concentrations had some degree of systematic bias. This suggests that, in some circumstances, it may be reasonable to compare cytokine concentrations in serum and plasma and to use samples that have undergone a thaw.

In this study we aimed to not only test and quantify the absolute relationships between samples, but also to accurately describe these relationships. We tested the differences between plasma and serum, plasma with and without a thaw, and serum with and without a thaw. Cytokines IL-2 and GM-CSF were only detectable in single samples, and these were not subjected to statistical analysis. Differences between absolute cytokine concentrations in serum and plasma were expected given the different biological properties of these media. However, as this study shows, these differences can be relatively small and predictable. 

At baseline, the ICCs suggested poor agreement between serum and plasma sample levels for IL-1*β*, IL-2, IL-4, IL-5, IL-6, IL-7, IL-8, IL-10, IL-12p70, IL-13, IL-17, G-CSF, GM-CSF, TNF-*α*, IFN-*γ*, MCP-1, and MIP-1*β*. Following the thaw condition, we found that only IL-1*β* had very good agreement (ICC = 0.90) in plasma. Even then, cautious interpretation is warranted, given the wide 95% confidence intervals (CI; 0.69–0.97). In serum samples, we found that the levels of IL-10 and IL-13 remained stable following the thaw condition. IL-7 also remained stable (ICC = 0.88) although again the 95% CIs were wide (0.62, 0.96). Using the Bland-Altman approach, we found that IL-1*β*, IL-5, IL-6, IL-8, and MIP-1*β* had moderately good agreement that was better at low concentrations of IL-1*β*, IL-4, IL-6, IL-7, IL-12p70, IFN-*γ*, TNF-*α*, and MCP-1 than at high concentrations. At low concentrations, the levels of these cytokines were similar in serum and plasma.

Following a thaw-freeze cycle, the values of all cytokines changed significantly in both serum and plasma. While the ICCs delivered poor absolute agreements, Bland-Altman analyses were used to further examine the relationships. Moderately good agreement was found for IL-1*β*, IL-8, IL-13, and MCP-1 in plasma and for IL-6, IL-7, IL-8, IL-10, IL-13, IFN-*γ*, TNF-*α*, MCP-1, and MIP-1*β* in serum. Good agreement was found at low concentrations of IL-4, IL-6, IL-7, IL-12p70, IFN-*γ*, TNF-*α*, and MIP-1*β* and higher concentrations of IL-5. No bias was detected in plasma samples. 

This study was subject to two main limitations. Firstly, although our sample was larger than in previous studies, it resulted in wide confidence intervals. Secondly, as we expected in an unmodified clinical sample, high proportions of out-of-range values were measured in our analytes. However, our main aim was to perform these analyses in samples obtained from a clinical population as it is possible that artificial procedures such as cytokine spiking could theoretically alter results. To address these shortcomings, future studies could obtain samples from disease populations where higher cytokine levels are predicted, such as from patients with systemic inflammatory conditions. Lastly, we found that some intra-assay CV%s were higher than 10% for IL-1*β*, IL-5, and GM-CSF. Perhaps more importantly, the low-concentration CV%s were high for IL-7 and IL-8. This suggests that we cannot be certain of the true magnitude of the agreements measured for these cytokines as some of the differences may be due to low assay precision. 

A major strength of this study was that samples were obtained from a clinical population using standard collection, processing, and storage methods. Some previous studies have prepared supraphysiological samples which may not reflect the behaviour of biological levels of cytokines in serum and plasma. 

While previous studies had found an effect of thaw-freeze on cytokine concentrations, those results concerned only a few cytokines and used simple measures of agreement. Although an agreement index is convenient, by also undertaking Bland-Altman analyses, we obtained a more complete picture for many cytokines. This highlights the benefit of a multiple analysis approach. In the present study we found that, in an absolute sense, cytokine concentrations were significantly affected by sample medium and by a thaw-freeze. However, given the additional information provided by the Bland-Altman analyses, we showed that moderately good agreement exists for several tested cytokines and that, at lower concentrations, excellent agreement was present. It is important to appreciate that the question of agreement should be answered in the context of need. For some clinical and research purposes, the level of precision found in this study may justify the direct comparison of serum and plasma. Similarly, the error resulting from the measurement of cytokine levels following a thaw may prove to be acceptable in certain situations. Significantly, as the difference between serum and plasma is a function of concentration for some cytokines, future research that compares these analytes should control for this change.

In conclusion, we found that the agreement between cytokine concentrations in serum and plasma is different depending on the cytokine of interest and can depend on its concentration. We identified that larger differences between samples exist at higher concentrations for some cytokines, suggesting that the data should be log-transformed to minimise the impact of this concentration-dependent effect. Thawing of samples changes the levels of cytokines considerably and should be avoided unless a moderate degree of error is considered acceptable.

## Figures and Tables

**Table 1:** Intraclass correlations (ICCs) and their 95% confidence intervals (CI); mean differences between the two measurements and their 95% confidence intervals; 95% limits of agreement which are expected to include 95% of the differences between the two measurements. Statistically significant values (*P* < 0.05) are denoted by bold font and *.

Cytokine	Baseline plasma versus baseline serum			Baseline plasma versus thawed plasma			Baseline serum versus thawed serum		
	ICC (95% CI)	Mean diff. (95% CI)	95% limits of agreement	ICC (95% CI)	Mean diff. (95% CI)	95% limits of agreement	ICC (95% CI)	Mean diff. (95% CI)	95% limits of agreement
IL-1*β*	0.16 (−0.27, 0.53)	−1.36 (−4.91, 2.19)	−17.45, 14.73	0.90* (0.69, 0.97)	−0.13 (−0.55, 0.29)	−1.43, 1.17	0.00 (−0.53, 0.53)	0.42 (−0.24, 1.09)	−1.73, 2.57
IL-2	—	—	—	—	—	—	—	—	—
IL-4	0.03 (−0.33, 0.42)	0.60 (−0.81, 2.00)	−5.62, 6.82	−0.15 (−0.667, 0.467)	−1.33 (−4.44, 1.77)	−10.41, 7.74	0.00 (−0.576, 0.576)	0.91 (−0.69, 2.50)	−3.74, 5.56
IL-5	0.14 (−0.25, 0.50)	−1.06 (−3.25, 1.12)	−10.73, 8.61	−0.41 (−0.92, 0.29)	−0.64 (−5.08, 3.81)	−13.60, 12.33	−0.11 (−0.72, 0.52)	0.01 (−2.70, 2.72)	−7.90, 7.91
IL-6	0.00 (−0.40, 0.39)	−1.91 (−20.69, 16.87)	−87.02, 83.20	0.41 (−0.13, 0.77)	7.07 (−11.18, 25.33)	−52.13, 66.28	0.00 (−0.60, 0.60)	−0.18 (−0.60, 0.23)	−1.32, 0.95
IL-7	−0.21 (−0.57, 0.21)	−12.91 (−39.43, 13.61)	−133.11, 107.28	0.70* (0.23, 0.91)	−0.64 (−19.54, 18.25)	−58.93, 57.65	0.88* (0.62, 0.96)	−5.66 (−16.43, 5.11)	−37.07, 25.75
IL-8	0.04 (−0.38, 0.44)	−7.36 (−25.36, 10.65)	−88.97, 74.25	−0.01 (−0.66, 0.61)	−7.52 (−30.58, 15.55)	−70.72, 55.69	0.55* (0.02, 0.85)	−20.28 (−45.39, 4.83)	−93.54, 52.98
TNF-*α*	−0.14 (−0.55, 0.31)	−20.37 (−78.00, 37.25)	−275.11, 234.36	−0.21 (−0.82, 0.46)	−7.34 (−45.92, 31.23)	−119.89, 105.20	0.17 (−0.47, 0.68)	−51.92 (−186.80, 82.95)	−445.41, 341.57
IFN-*γ*	0.07 (−0.36, 0.47)	−73.44 (−264.42, 117.53)	−917.68, 770.80	−0.09 (−0.64, 0.54)	19.86 (−19.26, 58.98)	−87.32, 127.05	0.00 (−0.60, 0.60)	11.59 (−14.63, 37.82)	−60.26, 83.44
IL-10	—	—	—	—	—	—	0.99* (0.98, 1.00)	−6.52 (−200.48, 187.43)	−604.84, 591.80
IL-12p70	0.19 (−0.22, 0.55)	19.11 (−15.21, 53.43)	−140.18, 178.40	0.28 (−0.31, 0.72)	29.81 (−37.43, 97.04)	−177.60, 237.21	0.58* (0.08, 0.85)	3.27 (−8.19, 14.74)	−32.09, 38.64
IL-13	0.00 (−0.43, 0.43)	2.30 (−0.92, 5.52)	−10.81, 15.41	0.00 (−0.60, 0.60)	0.66 (−5.48, 6.80)	−15.00, 16.32	0.96* (0.87, 0.99)	−0.31 (−2.00, 1.38)	−5.23, 4.61
IL-17	0.00 (−0.40, 0.40)	2.25 (−2.42, 6.92)	−18.90, 23.40	—	—	—	0.65* (0.14, 0.89)	34.66 (−42.57, 111.88)	−190.64, 259.96
G-CSF	0.18 (−0.13, 0.52)	58.53* (15.59, 101.46)	−121.29, 238.34	−0.09 (−0.71, 0.54)	−19.24 (−98.03, 59.54)	−249.10, 210.61	0.00 (−0.45, 0.54)	37.64 (−11.71, 86.98)	−106.33, 181.61
GM-CSF	—	—	—	—	—	—	—	—	—
MCP-1	0.10 (−0.32, 0.49)	−22.26 (−74.18, 29.66)	−257.59, 213.06	0.09 (−0.44, 0.60)	−28.19 (−78.02, 21.64)	−181.89, 125.51	0.50 (−0.02, 0.82)	−57.60 (−112.55, −2.65)	−227.12, 111.92
MIP-1*β*	0.38 (−0.03, 0.70)	−253.37* (−442.17, −64.57)	−1021.11, 514.37	0.20 (−0.52, 0.72)	−63.71 (−343.30, 215.87)	−829.75, 702.32	0.72* (0.25, 0.92)	−137.73 (−357.49, 82.02)	−739.84, 464.38
log MIP-1*β*	0.72* (0.29, 0.89)	**−0.62 (−1.07, −0.18)**	−2.44, 1.19	0.59 (−0.66, 0.90)	−0.28 (−0.98, 0.43)	−2.21, 1.66	0.63 (−0.49, 0.91)	−0.48 (−1.13, 0.16)	−2.24, 1.27

**Table 2:** Percentage of samples measured as below the assay detection limit or out of range in serum and plasma at the baseline condition. Each cell marked with “—” indicates that the analyte was not detectable in any sample. The intra-assay coefficient of variation (CV%) and the low concentration CV% are provided for each cytokine.

Cytokine	Intra-assay CV%	Low conc. assay CV%	Baseline			
			Plasma		Serum	
			Mean (SD) pg/mL	Out-of-range (%)	Mean (SD) pg/mL	Out-of-range (%)
IL-1*β*	11.8	0.0	2.73 (6.37)	76	2.83 (9.33)	88
IL-2	2.6	3.2	—	100	1.60 (7.85)	96
IL-4	7.9	2.6	2.38 (3.23)	40	0.89 (2.33)	72
IL-5	13.9	7.4	3.87 (7.07)	64	1.14 (3.85)	92
IL-6	7.9	8.3	13.72 (35.51)	76	270.63 (1197.25)	80
IL-7	9.9	20.1	37.06 (36.09)	96	51.57 (43.04)	92
IL-8	7.7	17.0	51.87 (25.11)	8	59.25 (33.93)	4
TNF-*α*	7.4	2.7	35.22 (62.85)	52	45.92 (125.44)	84
IFN-*γ*	6.1	5.2	68.07 (132.47)	64	134.82 (423.19)	80
IL-10	8.9	5.4	—	100	58.16 (201.74)	92
IL-12p70	6.4	3.8	36.89 (90.47)	40	22.42 (19.35)	4
IL-13	11.0	2.6	80.18 (341.62)	48	4.43 (10.02)	56
IL-17	5.1	2.9	3.21 (15.71)	96	25.08 (122.85)	96
G-CSF	7.3	0.0	87.77 (82.43)	40	25.72 (61.50)	68
GM-CSF	14.7	1.0	—	100	—	100
MCP-1	6.3	8.6	129.37 (82.22)	4	147.31 (94.57)	4
MIP-1*β*	5.4	5.2	397.88 (364.30)	12	650.01 (444.19)	12

## Appendices

### A. Bland-Altman Plots Plasma versus Serum

The middle line indicates the mean difference between the analytes, and the outer lines are the 95% limits of agreement (see Figure 1).

### B. Bland-Altman Plots Plasma Baseline versus Thaw Conditions

The middle line indicates the mean difference between the analytes, and the outer lines are the 95% limits of agreement (see Figure 2).

### C. Bland-Altman Plots Serum Baseline versus Thaw Conditions

The middle line indicates the mean difference between the analytes, and the outer lines are the 95% limits of agreement (see Figure 3).
